# Real-World Robot Evolution: Why Would it (not) Work?

**DOI:** 10.3389/frobt.2021.696452

**Published:** 2021-07-27

**Authors:** A.E. Eiben

**Affiliations:** Department of Computer Science, Vrije Universiteit Amsterdam, Amsterdam, Netherlands

**Keywords:** evolutionary robotics, evolution of things, reality gap, learning and evolution, simulations, triangle of life framework

## Abstract

This paper takes a critical look at the concept of real-world robot evolution discussing specific challenges for making it practicable. After a brief review of the state of the art several enablers are discussed in detail. It is noted that sample efficient evolution is one of the key prerequisites and there are various promising directions towards this in different stages of maturity, including learning as part of the evolutionary system, genotype filtering, and hybridizing real-world evolution with simulations in a new way. Furthermore, it is emphasized that an evolutionary system that works in the real world needs robots that work in the real world. Obvious as it may seem, to achieve this significant complexification of the robots and their tasks is needed compared to the current practice. Finally, the importance of not only building but also understanding evolving robot systems is emphasised, stating that in order to have the technology work we also need the science behind it.

## 1 Introduction

The main purpose of this paper is to examine the notion of robots that evolve and investigate if it is practicable. Considering it from a broader perspective, the issue is the feasibility of the nascent concept of the Evolution of Things. As outlined in Eiben et al. (2012); Eiben and Smith (2015a) artificial evolution can be positioned by the substrate where it takes place. The birth of Evolutionary Computing in the 20th century represented a major transition of evolutionary principles from wetware to software and the Evolution of Things amounts to a second transition from software to hardware. Regarding the type of evolvable objects one can distinguish passive artefacts, for example, industrial or fashion items, and active artefacts with agency, robots for short. Arguably, the latter type is more challenging and a key question is whether the evolution of real (not simulated) robots is different from the evolution of virtual organisms? Will robot evolution work by applying the methods developed for digital evolution or does it represent a whole new game where old tricks do not work, possibly spoiling the whole idea.

Obviously, there are arguments for as well as against the feasibility of robot evolution. On the positive side, one could say that artificial evolution of robots should work because natural evolution of living organisms has worked. That is, since we have convincing evidence that evolution is capable of developing adequate life forms for various environments we assume that evolution is capable of developing adequate robots for various applications. Admittedly, this may sound as wishful thinking, but there are also solid technical arguments. Specifically, 40 plus years of evolutionary computing has proved that evolutionary algorithms (EA) are capable of delivering high quality solutions to hard problems in a variety of scientific and technical domains, offering several advantages over traditional optimization and design methods Ashlock (2006); Bäck (1996); De Jong (2006); Eiben and Smith (2015b). One of the lessons learned is that EAs work in the face of problem characteristics that are very challenging for traditional approaches, for instance, the lack of analytical models, non-differentiability, discontinuities, multiple local optima, noise, and nonlinear interactions among the variables. To put it simply, when the going gets tough, the EAs get going.

On the negative side, there are good reasons to be sceptical about the feasibility of robot evolution. Specifically, our limited understanding of the intricate mechanisms underlying natural evolution Stanley and Miikkulainen (2003); Fernando et al. (2011); Cheney et al. (2016) and the billions of years it took to develop sophisticated life forms on Earth can raise doubts about replicating this in technological artefacts on a reasonable time scale. The first aspect concerns the deeper question whether it is necessary to emulate the evolutionary mechanisms as they work in natural organisms? Based on the achievements in evolutionary computing and evolutionary robotics it can be safely asserted that this is not necessary and implementing the *principles*, rather than the specific *mechanisms* is enough. The second aspect relates to the speed of evolution: If we can successfully implement selection, reproduction and heredity in a robot population, will that system produce high quality solutions in a few weeks or months, perhaps years, or will it take millenniums or longer. Technically speaking, this is the issue of sample efficiency and the core concern is that real world robot evolution will not work, unless made very sample efficient Hu and Banzhaf (2010).

Feasibility aside, one could also question the usefulness, that is the expected benefits of evolving robots. In other words, if the idea was feasible, why would we want to have such systems? The answer is twofold distinguishing theoretical and practical advantages. For scientific research, evolving robot systems can be considered as hardware models of evolving organisms useful as a research instrument to study hypotheses about evolution Long (2012). For practical purposes, they represent a (r)evolurionary engineering approach to develop robots for demanding applications. As a motivational example, consider the design of robots for monitoring rain forests in a remote region of Earth. Conventional engineering approaches can deliver good robot designs for not too complex, structured environments with predictable conditions that are known in advance and do not change over time. Rain forests do not fall into this category and there is no well-established prior knowledge regarding optimal robotic body forms for such environments. The space of all possible designs is big and complex, for instance, it is not clear whether a good robot should have legs, wheels or both? Should it be small and flexible navigating through holes in the vegetation or big and heavy to crush obstacles? And once an adequate body form is found, what is the appropriate control mechanism (brain) to drive it? These are questions that pose big challenges for traditional engineering; meanwhile these are the kind of challenges that evolution has solved successfully. One benefit of evolving robots is that evolution can deliver solutions to problems that are too hard for classic approaches. Additionally, evolution can deliver unexpected, original solutions that are out of the box for regular designers, see Hornby et al. (2011) for a great example. A third benefit is the inherent ability to adapt to changing conditions. Our current notion of machines is static with no or only very limited adaptivity. Evolution, however, is an adaptive force that can adjust bodies and brains of organisms if environmental changes require it.

In the long term I envision evolving robot systems become examples of what I call second order design or second order engineering. First order system engineering is the current practice where robots for a certain application are developed directly by humans. Evolutionary robot technology radically changes this picture because it introduces a new layer: instead of directly constructing a robotic system for a given application, humans are constructing an evolutionary system that will construct a robotic system. In my view this goes beyond using algorithms to support robot design; by the evolutionary approach as discussed here robots will develop themselves on the job.

## 2 State of the Art

To start the discussion let us note the difference between the concept of real-world robot evolution and the research area of evolutionary robotics as we know it. The field of evolutionary robotics has emerged in the 1990s and gained wide recognition from about 2000 when the first book fully devoted to the subject was published Nolfi and Floreano (2000). The broadly accepted description defines evolutionary robotics as the field of using evolutionary algorithms for designing and optimizing the overall morphology (body), the controller (brain) or both for simulated and real robots Bongard (2013); Vargas et al. (2014); Doncieux et al. (2015); Nolfi et al. (2016). Despite this general definition encompassing pretty much all possible options (disregarding sensors perhaps), the practice to date is quite limited: The huge majority of publications is in the digital realm about evolving the controllers for fixed bodies Radhakrishna Prabhu et al. (2018) and the few papers addressing morphological evolution, that is, the evolution of bodies, are almost all limited to simulations Auerbach and Bongard (2014); Bongard and Lipson (2014); Cheney et al. (2016), Cheney et al. (2017); Banarse et al. (2019); Miras et al. (2020); Gupta et al. (2021). This practice is in stark contrast with the concept of real robots reproducing and evolving in the real world.

Physically instantiated artificial evolutionary systems have been described and extensively discussed first in 2012 in Eiben et al. (2012). At that time there were no available technologies to create working implementations but the paper did outline the most important challenges and prerequisites:

1) Body types: An appropriate design space of all possible robot makeups.
2) How to start: A robot (re)production system to deliver physical robot offspring.
3) How to stop: A switch to stop evolution if necessary to prevent the “Jurassic Park problem”.
4) Evolvability and rate of evolution: The system must make good progress in real time.
5) Process control: A human, algorithmic or combined evolution manager to steer evolution.
6) Body-mind coevolution and lifetime learning: A period of infant learning to optimize the inherited brain for the inherited body quickly after birth.

Implementing such an evolutionary system is extremely challenging and as of to date this has never been realized. Nevertheless, a handful of systems have come close by demonstrating the simultaneous evolution of morphologies and controllers and using real robots in some way. One specific category of studies is based on the idea that the process of evolution is conducted in a simulator and only the result of this virtual evolution (a few good robots) is physically constructed, for example Lipson and Pollack (2000), Auerbach et al. (2014), and Kriegman et al. (2020). Such systems are obviously far from the envisioned real-world robot evolution and suffer from the infamous reality gap problem Jakobi et al. (1995); Mouret and Chatzilygeroudis (2017).

Among the existing systems where real world robots play a prominent role the first one is that of Long *et al.* investigating the evolution of vertebrae through swimming robot fish Long (2012); Cho (2014). This landmark project clearly demonstrated the potential benefits of robot evolution for fundamental research. In the meanwhile it also illuminated the practical problems, for instance, manual construction of new generations took days to weeks, which severely limited the number of experiments and the number of generations per experiment.

Another study on the morphological evolution of physical robots showcased a solution to challenge two above based on modular robot morphologies Brodbeck et al. (2015). Two types of cubic modules (active and passive) formed the raw materials and robot bodies were constructed by stacking and gluing a handful of such modules. The robot bodies were simple and they were not autonomous (driven by an external PC) and their task was to locomote. Robot genomes encoded the bodies implicitly by specifying the sequence of operations to build them by a robotic arm. The construction of new robots (“birth process”) was hands-free in some of the reported experiments, but required human assistance in some others.

The Robot Baby Project.^1^ was a proof-of-concept study to demonstrate robot reproduction, and implicitly robot evolution, in a real-world habitat Jelisavcic et al. (2017). While the system is simple, a unique feature is that robots coexist and can (inter)act in the same physical space where they can “meet and mate”, thus producing offspring. In the usual evolutionary robotics setup, including the systems in Long (2012); Brodbeck et al. (2015), a traditional EA performs evolution, where robots are manifested one by one and evaluated in isolation during the fitness evaluation step of the EA cycle.

The most recent development is taking place within the Autonomous Robot Evolution project.^2^ that is aiming at the construction of the first evolutionary system of autonomous mobile robots with complex body plans, sensors and controllers Eiben et al. (2021). The system is based on a Robot Fabricator, the RoboFab, to implement robot (re)production without human assistance Hale et al. (2019). The project is still running, but the experiences with an autonomous manufacture and assembly process revealed that real-world robot reproduction introduces new constraints on evolution that are not apparent in simulation Buchanan et al. (2020).

Before finishing this section let me mention two related areas with an interesting future potential for evolving robots in new substrates. Soft robots go beyond the currently dominant mechatronical designs by using soft materials that can facilitate new types of sensing and actuation and these can be combined with evolutionary approaches Rieffel et al. (2013); Laschi et al. (2016); Howison et al. (2021). However, to date such systems are either in simulation or are limited to “body parts”. The evolution of autonomous, untethered soft robots is still unfeasible. Another interesting development of late is formed by 4D-printed origami-robotic or soft robotic systems Hann et al. (2020); de Marco et al. (2018). This technology enables robots capable of dynamic morphological changes driven by environmental stimuli. Nevertheless, in their current form such systems are not evolutionary because there are no mechanisms for reproduction and inheritance.

In summary, the current state of the art is limited in two important ways. First, most studies rely on simulation to first evolve designs before constructing a physical robot. Systems where reproduction and evolution takes place in the real world are scarce. Second, the robot designs are usually very simple, robots contain only a few simple components, have no sensors and are driven by elementary open-ended control mechanisms that can not take environmental information into account. This implies that the behaviors these robots exhibit and the tasks they can solve are extremely simple, making any real-world counter part, a physical twin, practically useless.

## 3 Different System Types

Regarding the overall architecture, evolving robot systems can be divided into different categories by considering human involvement in the two principal components of evolutionary processes, selection and reproduction, as shown in Table 1.

**TABLE 1:** Different types of evolving robot systems depending on human involvement.

	Selection with humanAssistance (breeding)	Selection withoutHuman assistance
Reproduction with human assistance	Type 1	Type 2
Reproduction without human assistance	Type 3	Type 4

The need for human assistance in reproduction is rooted in the current lack of technology to 3D-print a fully functional robot at once. For instance, high quality motors and logic boards cannot be printed, and thus must be prefabricated and assembled with the 3D-printed components. Hence, human assistance may be necessary if a fully automated combination of 3D-printing and assembly line is infeasible.

Human involvement in the selection component of an evolutionary process is nothing new. Interactive evolutionary computation has been around for decades Takagi (2001) and human-driven selection is practiced for thousands of years by breeders of plants and domestic animals. For increased clarity it is useful to divide evolutionary selection into mate selection (parent selection in evolutionary computing) that determines what individuals can reproduce and propagate their genes and environmental selection (survivor selection in evolutionary computing) that determines what individuals survive and live long enough to reproduce. One could argue that in a robot system that evolves in the real world environmental selection is for free. However, for mate selection the users need to design and implement algorithms or play the selection mechanism themselves. Choosing the latter option, or a combination of algorithmic and human selection, can significantly increase the feasibility of creating and operating evolving robot systems.

Distinguishing these four types of evolving robot systems is relevant because this taxonomy shows that there exist important shortcuts making the implementation of evolving robot systems easier without invalidating the main principles: selection, reproduction, heredity. To illustrate the practical differences between these types of systems let us discuss examples for the two extremes, Type 1 and Type 4.

A system of Type 1 can be applied when evolution is used as a design method. In this case, the purpose of the evolutionary process is to find an optimal robot makeup for a given application in an off-line fashion. The process is halted if it delivers a robot that can operate in the given environment and performs well on the given tasks. This concludes the design process with the optimal robot as output and initiates the production process where many copies of this robot can be manufactured and deployed in the real-world. Based on the analogy with human farmers, such a setup can be seen as robot breeding. For the example of robots for inspecting rain forests this means to build a mock-up environment, for example, a garden, and to supervise the evolution of good solutions by monitoring and steering the evolutionary process.

In this example, and in most evolutionary computing applications, evolution is (ab)used as an optimizer that is halted when a satisfactory solution is found. Real evolution, however, is not about optimization, but about adaptation that never stops. This feature can be implemented through evolving robot systems of Type 4 allowing robot populations adapt to previously unknown and changing conditions on-the-fly without direct human oversight. This can be essential in hostile or inaccessible environments, like seafloors, cave systems or space. To illustrate the latter, imagine a mission for terraforming on another planet, where designing an optimal morphology and control system in advance is unfeasible. An evolutionary engine operating autonomously on the planet can mitigate this problem. Given that reproduction and selection are the two main forces behind evolution, an evolutionary engine should have two major components. The first component is a (re)production facility that can construct a large variety of robots. Depending on the specific circumstances and the applied technology, such a (re)production facility can make use of a repository of prefabricated components, an ‘organ bank’ that stores, for example, CPUs, servo motors, and cameras. Alternatively, it can utilize local resources, such as the gases in the atmosphere and the soil of the planet, and convert them into raw materials for the advanced 3D-printer that produces the new robots. The second component of an evolutionary engine is a twofold selection drive, such that robots become fit for the environment as well as fit for purpose. Environmental selection (for viability) is for free, as robots with a poor feature set will not be able to operate adequately. Mate selection, in turn, can be pre-programmed such that robots have a ‘basic instinct’ to chose mating partners with a high task performance (utility). The evolving robot population will then become increasingly adapted to the given planet and adjust their bodies and brains when the conditions change. Let us note that the terraforming application is closer to biological evolution, while a breeding farm is more like a usual evolutionary design process Bentley (1999).

## 4 Learning and Robot Infancy

Learning within an evolutionary process is a classic subject of studies in natural as well as artificial systems Smith (1987); Depew and Weber (2003); Nolfi and Floriano (1999). In the context of artificial evolution learning has been an optional feature with advantages and disadvantages Cecconi et al. (1996); Mayley (1997) but Eiben et al. (2013) argued that learning is a must for robots that evolve in real-time and real-space. Consequently, a generic system architecture underlying real-world robot evolution must inherently include a learning component. This stance was recently summarized in one sharp statement: *“If it evoles it needs to learn”*, cf. Eiben and Hart (2020).

**FIGURE 1:**
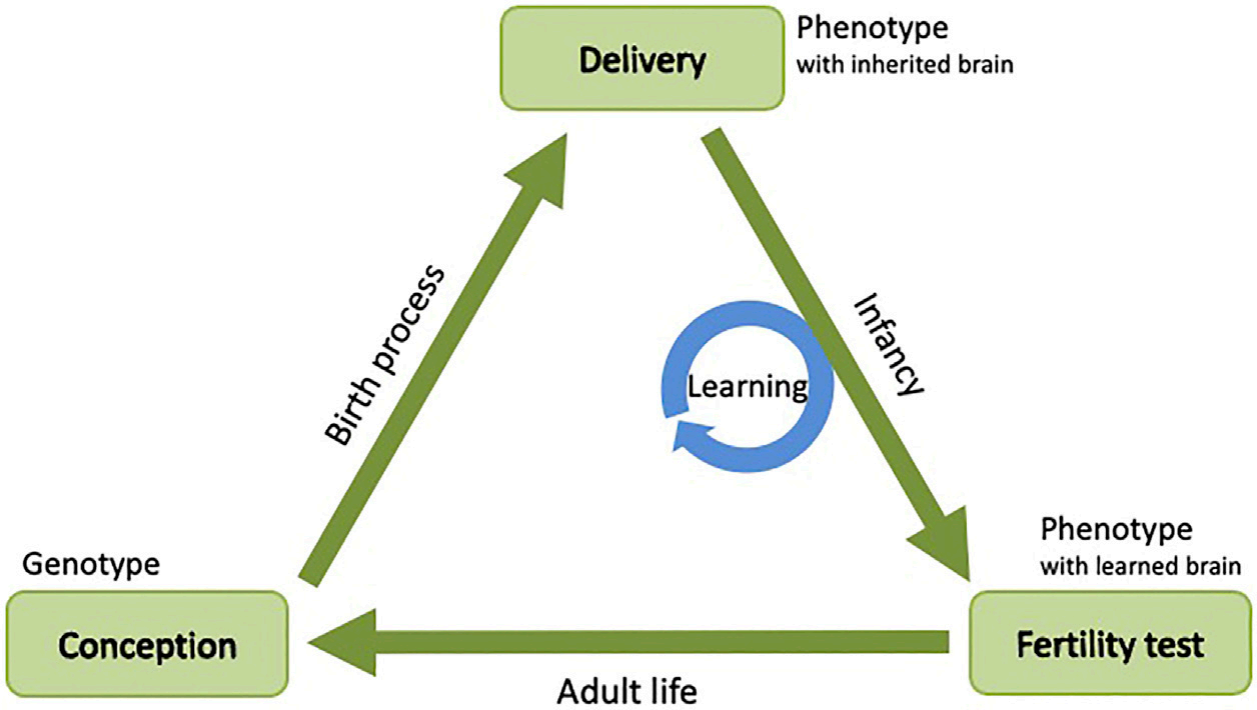
Generic system architecture for robot evolution conceptualized by the Triangle of Life, after Eiben et al. (2013).

This need for learning is rooted in the joint evolution of bodies and brains. In particular, by the stochastic reproduction of bodies and brains there are no generic guarantees that the inherited body and the inherited brain of a new robot match each other well. Even though the parent robots had well-matching bodies and brains (otherwise they would not have been fit enough to be selected) randomized recombination and mutation can result in a mismatch in the offspring. For instance, the body of the offspring may have actuators for which the inherited brain does not have appropriate control mechanisms. To mitigate this, a “newborn” robot must optimize its inherited brain quickly after “birth” such that it can adequately control the inherited body. Additionally, in the space of all possible brains for the ‘newborn’ body, the inherited brain is just one possibility. In other words, the evolutionary search operator (reproduction) only considers one sample in that brain space, leaving room for improvement. This improvement can be achieved by an additional search process in the brain space, making the robot smarter by finding a better brain for the inherited body. Therefore, the life span of a robot should not consist of two stages, the morphogenesis stage before ‘birth’ and the actual operating period after ‘birth’. Instead, it should consists of three stages: the morphogenesis phase, a learning phase, and the actual operational phase. This idea is conceptualized by the notion of the Triangle of Life as introduced by Eiben et al. (2013) and illustrated in Figure 1. This triangle captures the life cycle of evolving robots from conception (being conceived) to conception (conceiving their offspring) through three stages:

1) Morphogenesis: The process of creating a robot phenotype based on a genotype. For real-world robot evolution this is the toughest technical challenge, since it amounts to constructing a fully functional robot phenotype according to the specifications represented in a given genotype.
2) Infancy: The period when the “newborn” robot is learning to optimize its performance on a number of morphology dependent tasks or skills, such as locomotion, obstacle avoidance, terrain negotiation, and object grabbing.^3^ The infant learning process is concluded by an examination testing the robot’s performance and calculating its fitness. If the robot successfully passes this examination then it is declared a fertile adult and can start its mature life, otherwise it is removed from the system. An essential feature here is that the robot is not fertile (not eligible for reproduction) during the infancy stage.
3) Maturity: The stage when the adult robot operates normally, that is, tries to survive, performs its tasks, and reproduces, thus starting a new cycle. Naturally, robots can learn in this stage too, but as opposed to the supervised learning in the Infancy stage, for adult robots self-supervised or unsupervised learning is more appropriate.

The essence of the Triangle of Life is the designated learning stage, when the robot body does not change, but the brain driving it does because it undergoes a learning process. This learning process is in fact a search process through the space of all possible controller configurations to optimize the control of the inherited body and realize its maximum potential. It is important to note that in principle any search algorithm is applicable as a learning method, if only it can search in the space of possible controllers. Options to this end include Bayesian optimization, simulated annealing, reinforcement learning, and evolutionary algorithms.

**FIGURE 2:**
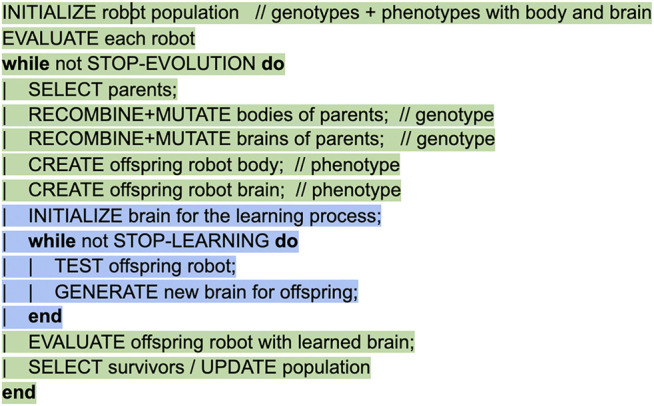
Pseudocode for robot evolution based on the Triangle of Life framework. Following the color code of Figure 1, the green lines correspond to the main evolutionary loop, the blue ones show the learning loop during the infancy stage. Note that the dichotomy of body and brain in the robots’ makeup translates to twofold genotypes divided into a body-coding segment and a brain-coding segment. These segments can have their specific recombination and mutation operators.

Using an evolutionary algorithm as a learning method represents a special case that leads to a double evolutionary system in a nested structure: an EA for lifetime learning (the blue circle in Figure 1) which optimizes a controller in a fixed body inside an outer evolutionary loop (the green triangle in Figure 1) which optimizes both body and brain. Obviously, the details of these two EAs may be completely different regarding the representation, mutation, recombination, and selection operators. From the evolutionary perspective of the outer loop selection is suspended during the Infancy stage, because fitness is only measured at the end after finishing the learning process as shown in the pseudocode of Figure 2. This property prevents reproduction of infant robots that have not demonstrated a good fitness (yet), hence it can save resources.

Interestingly, a system based on the opposite philosophy has also been proposed. Cheney et al. (2017) describe a morphological innovation protection mechanism which is in essence a variation of the Triangle of Life concept in that it allows additional optimization of the controller in a “newborn” body. By design, the authors do not apply specific learning operators to search through the space of brains, but use the mutation operator of their evolutionary algorithm. This is why controller optimization during the protected period is not perceived as learning, but as applying “several generations of evolutionary change restricted to the control subsystem”. Obviously, this is just a difference in perception and terminology, but the morphological innovation protection mechanism differs from the Triangle of Life also in a technical detail, because only survivor selection is suspended during the learning period and mate selection is not. In other words, during the protected Infancy period a robot can produce offspring, but it cannot be selected for removal from the population. In this respect the method is based on different priorities: Instead of protecting the population from being contaminated by the (possibly inferior) genes of a new individual, it protects a new individual from being wiped out by the (possibly superior) population members. An empirical study comparing these approaches is ongoing.

Obviously, the Triangle of Life framework is not restricted to being used for real-world robot evolution. A system with a specific Infancy stage can just as well be applied in simulation. However, I argue that while for simulated systems it is a *neat-to-have*, for real-world robot evolution it is a *need-to-have*. The reason is that in a real-world application learning forms a relatively fast and cheap way of reducing the number of slow and expensive evolutionary steps. To be specific, we can distinguish learning trials and evolutionary trials. An evolutionary trial equals to producing, activating and testing a new robot, including the manufacturing of its body, while a learning trial means producing, activating and testing a new controller in a given robot. Because controllers are digital entities, just pieces of code, learning trials cost much less time, effort and material resources than evolutionary trials.

For the proverbial calculation on the back of an envelope let us estimate the time of manufacturing a new robot by 12 h, the time needed for computing and installing a new controller to be tried by 10 s and let us set the duration of one fitness evaluation at 1 minute. (For the sake of the argument assume that these numbers are independent from the robot morphologies.) Then 1,000 evolutionary trials using one (re)production facility need approximately 501 days, while 1,000 learning trials cost less than 1 day. This emphasizes the need for sample efficient evolution that does not need to generate and test many robots to achieve good performance. To this end, the role of learning is to maximize the performance of a given robot by acquiring a top-quality controller that realizes the full potential of the given morphology.

Fortunately, the idea of designing controllers for robots through using machine learning techniques is gaining traction independently from evolutionary robotics Hwangbo et al. (2019); Lipson (2019). Methods developed in that field are likely to be applicable in combination with evolution, although the evolutionary context poses an extra challenge: In the current practice the robot morphology is known to the developers and this knowledge can be exploited to customize and optimize the learning method, whereas in an evolving robot system “newborn” robots are not known in advance. This requires robust learning methods that work independently from the given robot morphology.

## 5 Exploiting the Artificial

The history of evolutionary computing in general and of evolutionary robotics in particular shows an ambivalent attitude with respect to copying tricks from natural evolution. On the one hand, the very idea behind both fields is to mimic Nature’s solutions and implementations of natural mechanisms in an artificial substrate appears to have an intrinsic value for many researchers. On the other hand, many evolutionary mechanisms need to be oversimplified to be transferable from wetware to software which can create quite a gap between natural and artificial evolution. This section argues and demonstrates that unnatural elements in an evolving robot system need not be a bad thing, on the contrary, they can be instrumental to make the system work. The main message is to embrace the artificial and gladly exploit the possibility to do tricks that do not exist in natural evolutionary systems.

### 5.1 Genotype Filtering

One of the crucial differences between the evolution of life on Earth and the evolution of robots is the scale. This concerns the population sizes as well as the affordable number of generations. To put it simply, natural evolution is extremely wasteful (produces large numbers of organisms many of which die before they could reproduce) and has all the time in the world, while practical evolutionary robot systems do not have the luxury to waste robots and time. This means that every newly created robot must count, that is, it should have the potential of being good and providing valuable information to steer the evolutionary process towards superior solutions.

For a better perspective, let us note that evolutionary computing and robots evolving in simulation are much less sensitive to such constraints than robots that evolve in the real-world. The reason is obvious, physical resources are much more scarce than digital ones. Rendering and activating a new robot in a simulator is way faster and cheaper than manufacturing one and activating it in the physical environment. Furthermore, computational resources are easier to scale-up by more powerful computers or bigger clusters. Thus, we are facing an issue that would make real-world robot evolution not work.

The solution to mitigate this issue is to use an unnatural trick: a sophisticated assessment of a new robot genotype (created by recombination and mutation of the genotypes of existing robots) before it is transformed into a new phenotype, that is, before starting the construction of the corresponding physical robot. A recent study introduced two notions to this end: manufacturability and viability Buchanan et al. (2020). Manufacturability is the property that a certain body plan can indeed be constructed with the given system for robot (re)production. This property is inherently practical, it does not only reflect whether the body plan is physically possible, for example, has no overlapping components, but also whether it can be built with the given machinery. Relevant details for the latter include possible overhangs in the parts that need to be 3D-printed and the geometry of the robot arms that are putting together the components that make up the complete robot body. Viability is a more generic notion in the sense that it applies to not only physical but also to virtual robots. A robot is considered viable if it satisfies the minimal requirements for being able to operate at all. For example, a robot (regardless whether it is manufacturable) may not be viable if it has no sensors or no actuators or battery.

Manufacturability and viability are two examples of a more generic notion of a genotype filter. In general, a genotype filter an observable or computable property of the genotypes that can be used to distinguish desirable and undesirable ones. Real-world robot evolution systems can and should apply such filters and prevent wasting resources by letting through only those genotypes that are worth being instantiated as physical phenotypes. Further to the practical relevance of such filters they raise intriguing questions about the influence of the (re)production mechanisms on evolutionary pathways, the accessible regions of the evolutionary search space, and ultimately on the practically accessible optima of the given design problem.

### 5.2 Hybrid Evolution: Two Populations, One Species

As explained in Long (2012) a simulated evolutionary robot system can be implausible in two different ways, biologically or physically implausible. The main message of *Exploiting the Artificial* is that in the context of evolving robots for practical applications we do not need a biologically plausible setup. The fact that a certain mechanism does not exist in living organisms does not make it useless. Physical plausibility is a different matter because of the reality gap. If a simulation violates the laws of physics, then its practical use if very limited. However, this does not mean that simulations are useless in a real-world evolutionary robot system. There are several studies in the literature that evolve robots in simulation and during the evolutionary process occasionally construct a robot in the real world to validate the actual behaviour and thus the real fitness of the given design Koos et al. (2013); Mouret and Chatzilygeroudis (2017).

The notion of such a mixed evolutionary system where all robots are evaluated in simulation and some robots also in the real world can be taken even further. The concept of a hybrid evolutionary system as outlined by Howard et al. (2019); Eiben et al. (2021) is based on a deeper integration between simulated and real evolution. The idea is to simultaneously evolve a simulated and real robot population where a real robot does not necessarily have a digital twin and vice versa. However, the two populations are deeply integrated by considering them as one species where any two individuals can produce offspring. A key feature to this end is using the same genetic representation in both worlds. This means that the set of syntactically correct genotypes is the same for the physical and the virtual robots. This enables cross-breeding so a new robot can have physical or virtual parents, or a combination of both. The inverse side of this property is that a newly created genotype can be instantiated in the physical world, in the simulator or both. Additionally, using the same genetic representation enables transferring robots between environments simply by sending a genotype from one environment to the other and applying the appropriate morphogenesis protocol to create the corresponding phenotype, a virtual or a physical robot.

The advantages of such a combination are straightforward. Physical evolution is accelerated by the virtual component that can find good robot features with less time and fewer resources than physical evolution, while simulated evolution benefits from the influx of genes that are tested favourably in the real world. In an advanced system, the physical trials can help improve the accuracy of the simulator as well, thus reducing the reality gap.

## 6 How to Make it Work

The science and technology of robot evolution is in an embryonic stage which implies that much needs to be researched and developed. Discussing all options is beyond the scope of this paper, so let us mention only three lines of research here: more realistic robots that work in the real world, sample efficient evolution and learning, and instruments to formally describe and analyse relevant properties of evolutionary robot systems.

To have an evolutionary system that works in the real world we need robots that work in the real world. Compared to the current practice in evolutionary robotics this requires (at least) the following three improvements. Firstly, robots with sensors and closed loop controllers that can take sensory inputs into account should be used. This is not only an engineering challenge, but also an extension of the scientific and conceptual framework from evolving body plans and brains to evolving body plans, sensors, and brains Tapia et al. (2020); Ferigo et al. (2021). Secondly, robots should be evolved for more and more practical tasks. In the current evolutionary robotics literature robots are usually evolved for one task that is very simple, for example, locomotion in an empty arena or navigation in a small maze. Real robots must posses multiple basic skills that can be used when performing more complex tasks. Practical skills include simple locomotion (gait), targeted locomotion (homing), object following, negotiating different terrains, obstacle avoidance, object grabbing, object transporting, just to name a few. Complex tasks, for example, exploring an environment and counting all red objects or going to a given area and collecting soil samples, can be performed only if the robots possess an adequate set of basic skills. Importantly, many of these skills is morphology dependent, hence each “newborn” robot with a novel morphology needs to acquire them anew. Thirdly, the evaluation of robot behaviour needs to be automated. This is essential to automate the learning process during the Infancy period and to determine fitness in the population of mature robots without human assistance. To this end, it is helpful to distinguish the robots’ performance regarding the basic skills learnt during the Infancy period and the performance considering the more complex tasks they are supposed to perform. In the long run–for Type 4 systems–autonomous reproduction needs to be accompanied by autonomous selection without a human in the loop.

Having a working evolutionary robot system with autonomous reproduction and autonomous selection is an ambitious goal, but still not enough for practical purposes if it is too slow Hu and Banzhaf (2010). To increase the sample efficiency of robot evolution, human assistance, genotype filtering, and hybridization with simulation as discussed before can be used. Furthermore, the existing knowledge developed in evolutionary computing offers several other tricks. For instance, multi-parent reproduction is known to speed up evolution Eiben (1999) and the use of surrogate models can reduce the time of fitness evaluations Dutta et al. (2020). Additionally, learning can act as an accelerator for evolution because improving the brain in a given body is relatively cheap in terms of resources and time, while it can significantly improve the robots’ fitness. A further advantage of learning is known as the Baldwin effect Baldwin (1896); DeJager (2016) that is somewhat controversial in biology, but demonstrated to occur and cause a useful effect in artificial evolutionary systems of different types French and Messinger (1994); Whitley et al. (1994); Aguilar et al. (2019); Valdivieso et al. (2006). Recently it has been shown that in morphologically evolving robot systems combined with learning the learning ability of the evolving morphologies is increasing over generations Miras et al. (2020); Gupta et al. (2021). Furthermore, results with a Lamarckian combination of evolution and learning where learned properties of controllers can be coded back to the robots’ genotypes, are very promising Jelisavcic et al. (2019). Ultimately, the learning methods can be also be evolved, making the transition from evolution *and* learning to the evolution *of* learning Soltoggio et al. (2018).

The third line of research I recommend concerns instruments to formally describe and analyse evolutionary robot systems. To this end, one can use methods from the theory of evolutionary computation Doerr and Naumann (2020) or borrow from the toolkit of evolutionary biology Freeman and Herron (2015); Futuyma and Kirkpatrick (2017). However, evolution in a robotic context represents new challenges and opportunities compared to these fields. Compared to usual EC, evolving robots induce more complex notions of phenotype and fitness. Robots are complex entities with bodies and brains embedded in time and space and their fitness is determined by their behavior (which in turn depends on complex interactions of their body, brain, and the environment), evaluated by a given set of tasks. Analysing such a system is arguably harder than describing the behaviour of an evolution strategy on a synthetic fitness landscape defined by a numerical optimizaton problem. Compared to biology, the robotic context offers extra options for analysis and experimentation, because the bodies, the brains and the behaviours of the robots are more programmable, controllable, and observable than those of natural organisms. A good angle to observing and tracking system dynamics is to look into the three main search spaces that correspond to the bodies, the brains, and the behaviours of robots. For instance, it is possible to define quantifiable morphological traits reflecting the dimensions, the geometry, or the symmetry of the robots’ body and use these for statistical analyses and insightful visualisations Miras et al. (2018). Similarly one could identify structural and functional properties that capture relevant aspects of the robot brain and define a controller trait space, for instance, by the density of the neural network or the periodicity of a given signal. While these are just a few examples, in general such tools will enable systematic studies on system parameters and the induced dynamics, the interactions of various components, like the body and the brain, and likely deliver useful knobs to steer the outcome of a complex evolutionary process. The long term relevance of this line of research is significant, because constructing and operating evolutionary robot systems will be impossible without understanding them.

To conclude, let me recall the main subject of this paper: physically implemented evolutionary robot systems. This brings up the notion of EvoSphere and the issue of controllable robot reproduction as introduced and discussed in Eiben (2015). An EvoSphere stands for an evolutionary robot habitat based on the three stages in the Triangle of Life, morphogenesis, infancy, and mature life. Hence, an EvoSphere consists of three components, the Robot Fabricator, the Training Facility and the Arena that represents the outside world where the mature robots need to operate. The Robot Fabricator is the unit where new genotypes are converted into new phenotypes, that is, where “newborn” robots are constructed. With the current technology, this is possible by using a combination of 3D-printers, a set of prefabricated modules (an “organ bank”), and automated assembly by industrial robot arms.

It is important to note that, while the Triangle of Life is neutral about the actual system for morphogenesis, there is a principal decision behind the EvoSphere concept. Specifically, an EvoSphere contains a designated system component to implement robot (re)production. This is not only a pragmatic engineering solution, but a deliberate design choice for the sake of ethics and safety. The underlying motivation is to reject robotic equivalents of cell division, laying eggs or pregnancy because such mechanisms could enable robots that can reproduce anywhere without control. In general, I argue that all forms of distributed reproduction systems should be avoided, including self-assembly of new robots consisting of autonomous components Kernbach et al. (2009); Levi and Kernbach (2010). Instead, for reasons of safety, a real-world robot evolution system should based on centralized reproduction, implemented by one Robot Fabricator.^4^ From the perspective of the robots, this is a single point of failure, from the human viewpoint, this is a safety switch. Shutting down the Robot Fabricator can stop robot reproduction, hence robot evolution, if necessary. The rationale is obvious, runaway evolution in a computer can only do limited harm, such as memory overflow or computer crash, but the consequences in the real world can be much more severe. I consider this an important issue and emphasize that all physically embodied evolutionary robot systems must be designed with a shutdown option to prevent the ‘Jurrasic Park problem’ as stated in Eiben et al. (2012).

**FIGURE 3:**
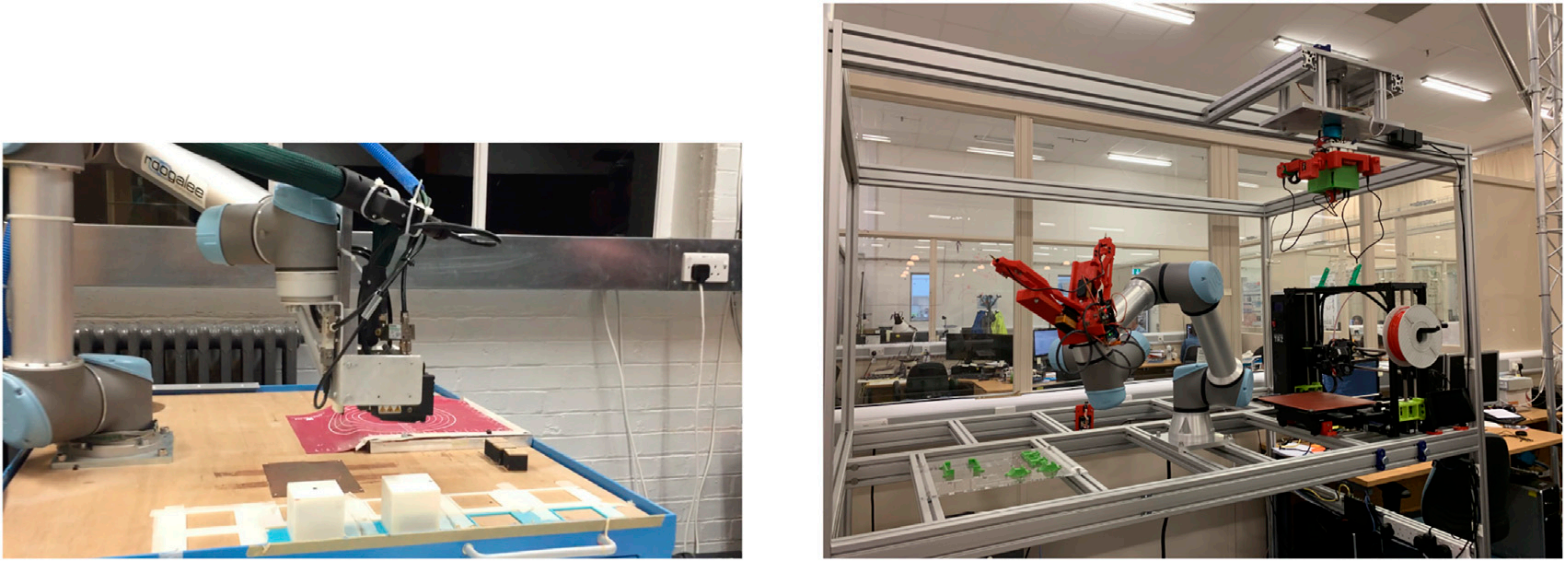
Two examples of a working Robot Fabricator. **(A)** The system in Cambridge, cf. Brodbeck et al. (2015). **(B)** The system in Bristol, cf. Hale et al. (2019).

Figure 3 shows the two examples of Robot Fabricators I know of at the moment. To my knowledge, currently there are no working examples of Training Facilities, where “newborn” robots learn using feedback from a computer vision system or a human user or both. However, considering the advances in material science, rapid prototyping, automated assembly, and the increasing interest in embodied intelligence and machine learning, I expect to see working Robot Fabricators and Training Facilities integrated with a test ground that serves as the Arena within five to 10 years from now.

## Footnotes

1 https://www.youtube.com/watch?v=BfcVSb-Q8ns.

2 https://www.york.ac.uk/robot-lab/are/.

3 Tasks that do not depend on morphologies need not be included here as they can be hard coded in all robots. For example, image processing or wireless communication abilities will not depend on the specific robot makeup and can be installed on all “newborn” robots regardless the given body form.

4 Of course, two or a some small number of Robot Fabricators is also acceptable, as long as they can be controlled and shut down if needed.
